# Controlled self-assembly of chemical gardens enables fabrication of heterogeneous chemobrionic materials

**DOI:** 10.1038/s42004-021-00579-y

**Published:** 2021-10-14

**Authors:** Erik A. B. Hughes, Thomas E. Robinson, Richard J. A. Moakes, Miruna Chipara, Liam M. Grover

**Affiliations:** 1 School of Chemical Engineering, University of Birmingham, Birmingham, B15 2TT UK; 2 NIHR Surgical Reconstruction and Microbiology Research Centre, Queen Elizabeth Hospital, Birmingham, UK

**Keywords:** Bioinspired materials, Self-assembly

## Abstract

Chemical gardens are an example of a chemobrionic system that typically result in abiotic macro-, micro- and nano- material architectures, with formation driven by complex out-of-equilibrium reaction mechanisms. From a technological perspective, controlling chemobrionic processes may hold great promise for the creation of novel, compositionally diverse and ultimately, useful materials and devices. In this work, we engineer an innovative custom-built liquid exchange unit that enables us to control the formation of tubular chemical garden structures grown from the interface between calcium loaded hydrogel and phosphate solution. We show that systematic displacement of phosphate solution with water (H_2_O) can halt self-assembly, precisely control tube height and purify structures in situ. Furthermore, we demonstrate the fabrication of a heterogeneous chemobrionic composite material composed of aligned, high-aspect ratio calcium phosphate channels running through an otherwise dense matrix of poly(2-hydroxyethyl methacrylate) (pHEMA). Given that the principles we derive can be broadly applied to potentially control various chemobrionic systems, this work paves the way for fabricating multifunctional materials that may hold great potential in a variety of application areas, such as regenerative medicine, catalysis and microfluidics.

## Abstract (web summary)

Chemobrionic materials with applications in tissue engineering can be grown at the interface of calcium and phosphate solutions, however, controlling their dimensions and purity can be difficult. Herein, a flow device allows control of the height and in situ purification of chemical gardens, which can be encased in a photoset hydrogel for subsequent use.

## Introduction

Chemobrionic systems encompass a diverse range of complex self-organisation reactions that typically result in the formation of hierarchical architectures. Chemical gardens are a classical example of a chemobrionic system first reported several centuries ago in 1646^1^. These formations are commonly characterised by the spontaneous formation of colourful hollow tubular precipitates following the introduction of metal salt seeds, which provide a broad spectrum of cations (e.g. calcium (Ca^2+^), copper (Cu^2+^), iron (Fe^2+/3+^), etc.), to reservoirs of anionic solution (e.g. carbonate (CO_3_^2−^), phosphate (PO_4_^3−^), silicate (SiO_4_^4−^), etc.)^2^. Structures can also be grown from the controlled injection of reactant solutions into one another, as well as from the interface between reactant-loaded hydrogels and solution reservoirs^3–6^. Though completely abiotic, the resulting structures often possess striking resemblances to biotic organisms and forms, including plants and fungi.

The understanding of fundamental chemobrionic principles has advanced significantly in recent decades, informing potential mechanisms of biomineralisation and pathways responsible for the formation of geological structures and artefacts^7–9^. In particular, the study of chemobrionics in relation to hydrothermal vents has helped to shed light on the origins of life^10–12^. Investigations simulating hydrothermal conditions have subsequently implicated the role of chemobrionic phenomena in providing energy and catalysing prebiotic chemical reactions, including the formation of energy-rich pyrophosphate species (P_2_O_7_^4−^), amino acids and ribonucleic acid (RNA)^13–16^.

From a technological perspective, chemobrionic reactions offer a means to build complex structures across nano-, micro- and macroscales from a plethora of organic and/or inorganic, ionic and/or molecular building blocks. Advances have been made in numerous areas of application, including but not limited to regenerative medicine^17^, biochemical and therapeutic delivery^18^, soft bioinspired materials^19^, catalysis^20^, electrochemistry^21^, gas sensors^22^, luminescent materials^23,24^ and microfluidics^25^. Harnessing control over chemobrionic processes may hold great promise toward the creation of novel, compositionally diverse and ultimately, useful materials and devices^26,27^.

Considering that thermodynamic disequilibrium drives self-organisation in many chemobrionic systems (excluding mechanically controlled injection systems), tubular chemical garden structures can continue to lengthen until the system either reaches a state of thermodynamic equilibrium or is depleted of reactant. Problematically, the continued deposition of precipitate in these confined reactions can result in the growth of structures that reach the solution–air interface, proceed to agglomerate and eventually, collapse. As such, the retrieval of intact frameworks requires structures to be harvested from the growth environment whilst still actively undergoing formation. Additionally, harvesting of structures can lead to loss of alignment and breakage. Therefore, the growth and acquisition of chemobrionic structures in such a manner that allows for ease of further processing toward technologically relevant materials remains highly challenging.

Calcium phosphate chemical gardens grown from hydrogel–solution interfaces have been proposed to hold great potential in the field of tissue engineering as cellular scaffolds and biologically responsive structures^28–30^. Self-assembly of calcium phosphate chemical gardens from the interface between a hydrogel and solution occurs in a mechanistically similar manner to the formation of architectures from cationic salt seeds submerged in anionic reservoirs^6,31^. In the hydrogel–solution system, calcium ions are homogeneously diffused throughout the hydrogel. A variety of hydrogel networks can be utilised, including agar, gelatin or carrageenan^6,32–34^. When phosphate solution is introduced on top, a semi-permeable precipitate membrane forms from the interaction of calcium ions at the surface of the hydrogel with phosphate ions in solution. Osmotic pressure differences between the outside and inside of the membrane draws water inward, causing the hydrogel to swell. The membrane also facilitates steep chemical and pH gradients that drive the interaction of reactive species.^29,35^ Eventually, the osmotic pressure causes the semi-permeable membrane to rupture, releasing streams of buoyant calcium-rich fluid into the surrounding anionic solution. Precipitation around these streams leads to the formation of calcium phosphate tubes. Repetitive build-ups in osmotic pressure and release maintains the ascension of calcium-rich fluid through established structures. Subsequently, the tips rupture and the emission of calcium-rich fluid leads to further precipitation, continuing the elongation of the tubes. In an uncontrolled set-up, calcium phosphate tubes can reach the solution–air interface and continue to grow along it. Agglomeration of tubes through the continued deposition of calcium phosphate will eventually lead to the collapse of structures, limiting processability.

The aim of this study is to determine a method to control the formation of calcium phosphate chemical gardens and take critical steps toward the development of a new class of biomaterial consisting entirely of, or incorporating, biologically relevant chemobrionic components. We engineered a custom-built liquid exchange unit that enabled us to investigate whether the systematic displacement of phosphate solution with water (H_2_O) could act as a means to control the self-assembly of calcium phosphate chemical gardens. Not only could we halt the growth of calcium phosphate tubes, we were also able to precisely control tube height and purify the structures in situ. Moreover, the integration of calcium phosphate tubes in a matrix of poly(2-hydroxyethyl methacrylate) (pHEMA) is demonstrated, highlighting a viable route for the development of novel chemobrionic materials for biotechnological applications.

## Results and discussion

### Interaction between phosphate solution and H_2_O

To explore the systematic exchange of phosphate solution with H_2_O as a means to influence calcium phosphate chemical garden growth, we engineered a custom-built liquid exchange unit by modifying a 0.14 L capacity Really Useful Box^®^ with inlet and outlet channels to enable the exchange of liquids as driven by external pumps. This allowed for H_2_O (or other liquids) to be controllably introduced in place of phosphate solution during self-assembly of chemical garden structures. Initially, the inlet channel was served by a reservoir of H_2_O, whilst the outlet channel led to an outlet reservoir to collect displaced phosphate solution.

**Fig. 1:**
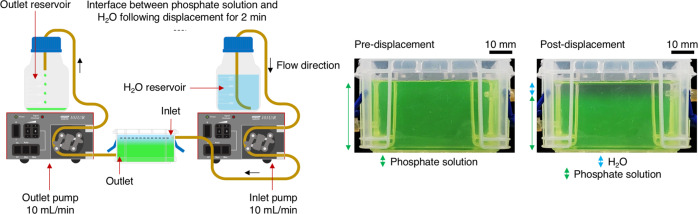
Displacement of phosphate solution with H_2_O. Phosphate solution was labelled with fluorescein to make it appear green in colour in contrast to clear H_2_O. Density forces were found to drive separation of the miscible liquids, resulting in the establishment of a layer of H_2_O on top of the phosphate solution.

We first sought to understand the interaction between phosphate solution and H_2_O (Fig. 1). To better visualise the interactions between the two liquids, phosphate solution was firstly labelled with fluorescein, allowing it to be distinguished from the incoming flow of clear H_2_O. Displacement of phosphate solution with H_2_O was performed at 10 mL/min for 2 min, resulting in the formation of a contrasting clear layer on top the of the phosphate solution. Given the likeness of this phenomena to an oil on water interface, we postulated that separation may be driven by buoyancy forces. In the context of liquid–liquid systems, a similar separation phenomenon is observed when a body of sea water of high salinity meets fresh ground water. Acute density differences between two miscible liquids can lead to buoyancy-driven separation^36,37^. Indeed, the density (ρ) of H_2_O (ρ = 1.01 ± 0.01 g/cm^3^ at 25 °C and 1 atm) was found to be measurably less than that of phosphate solution (ρ = 1.08 ± 0.01 g/cm^3^ at 25 °C and 1 atm). Moreover, phosphate anions in solution can possess attractive intermolecular interactions facilitated by short-range hydrogen bonding, which may further contribute to preventing immediate equilibration^38^. Only after several hours were the miscible liquids seen to completely equilibrate, suggesting that the separation of H_2_O and phosphate solution is stable over a period of time sufficient to influence chemical garden formation (i.e. halt growth).

### Controlled formation and purification of chemical garden structures

**Fig. 2:**
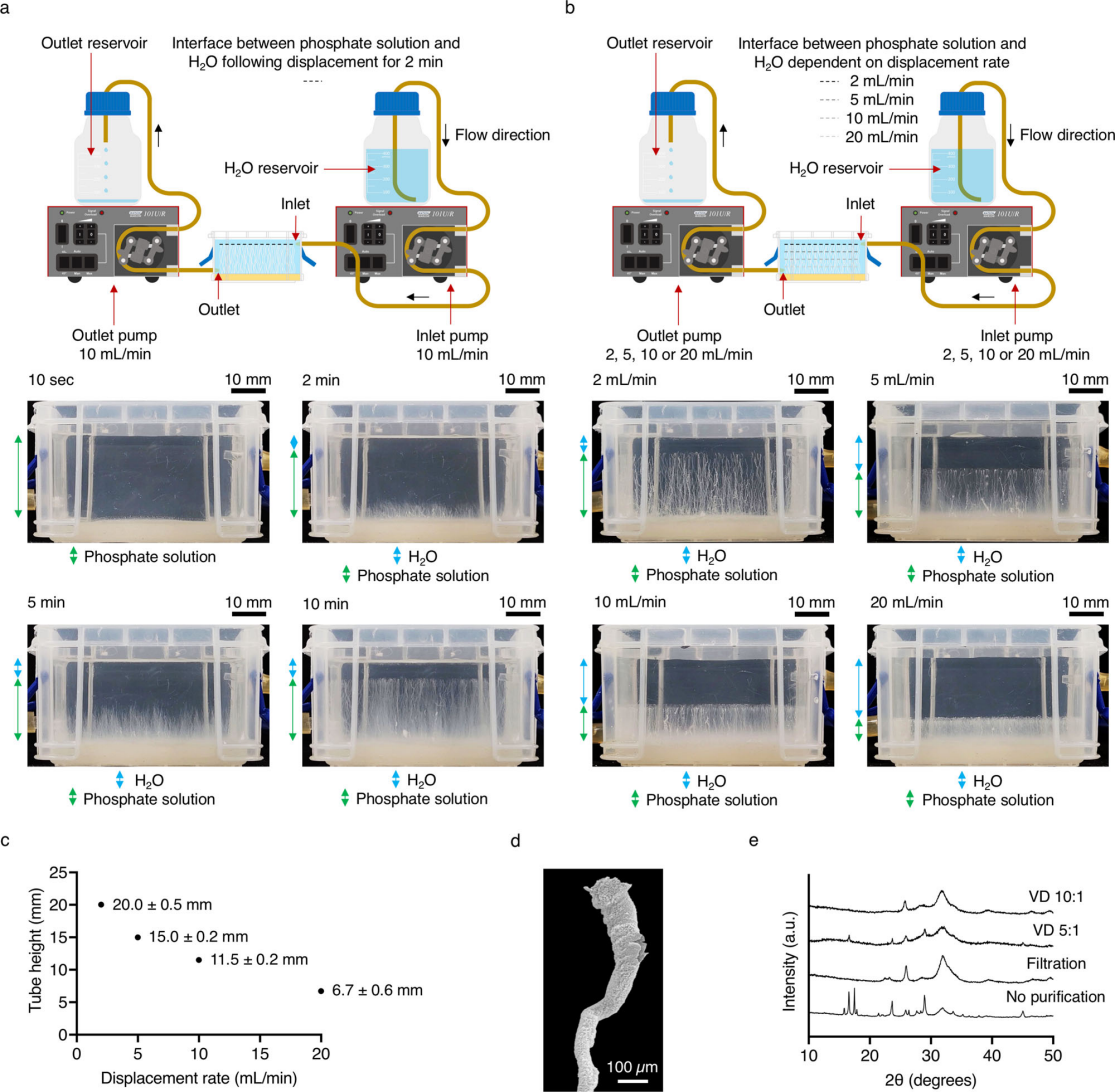
Controlled formation and characterisation of chemical garden structures. **a** Displacement of phosphate solution with H_2_O performed during the formation of calcium phosphate tubes. Tube growth was unable to proceed beyond the established H_2_O layer, demonstrating an effective means to uniformly halt self-assembly in situ. **b** Displacement of phosphate solution with H_2_O performed during the formation of calcium phosphate tubes at various displacement rates. Displacement was halted when the tips of the structures met the established H_2_O layer, resulting in tubes of with different heights. **c** Relationship between calcium phosphate tube height and displacement rate, demonstrating a high degree of reproducibility over chemical garden formation; error calculated using standard deviation (SD). **d** Scanning electron microscopy (SEM) image of a growth halted calcium phosphate tube, revealing a distinct dome shaped tip morphology; scale bar = 100 µm. **e** A comparative series of X-ray diffraction (XRD) patterns revealing compositional differences between structures treated without purification (No purification) and purified by filtration with excess H_2_O (Filtration), as well as structures purified in situ with a volume displacement (VD) ratio of either 5:1 or 10:1 (H_2_O to phosphate solution) prior to harvesting and subsequent characterisation.

To demonstrate the feasibility of controlling calcium phosphate chemical garden growth, displacement of phosphate solution with H_2_O was performed during the formation of calcium phosphate tubes from a hydrogel–solution interface (Fig. 2a). A layer of calcium-loaded hydrogel was firstly cast into the liquid exchange unit before introducing phosphate solution on top to initiate the self-assembly of tubular calcium phosphate structures. Displacement of phosphate solution with H_2_O was performed 2 min after the layering of phosphate solution over the hydrogel at a rate of 10 mL/min for 2 min. Structural elongation was unable to proceed beyond the interface between H_2_O and phosphate solution, resulting in an array of uniformly capped, aligned and intact calcium phosphate tubes after approximately 10 min. Had the structures continued growing toward the solution–air interface, the continued deposition of mineral would have led to the agglomeration and collapse of the structures. Normally, structures would require harvesting before this point to prevent this.

We then explored employing specific displacement rates to control tube height (Fig. 2b). Displacement of phosphate solution with H_2_O was performed 10 s after the layering phosphate solution over the hydrogel at rates of 2, 5, 10 and 20 mL/min. Displacement was halted when the tips of structures met the interface between phosphate solution and H_2_O. Through this process, calcium phosphate tubes of specific heights could be generated reproducibly, demonstrating a level of control over chemical garden formation (Fig. 2c).

For the displacement rates employed, the corresponding Reynolds number (Re) values were in the range of approximately 15–160 at the point of liquid entering the liquid exchange unit, indicating laminar flow during exchange (laminar where Re < 2000, transient where 2000 < Re < 4000, turbulent where Re > 4000)^39^. Maintaining a laminar liquid displacement regime did not appear to impede or disrupt the formation of structures compared to uncontrolled growth. Our observations, therefore, suggest that laminar flow of incoming fluid, together with density forces and attractive molecular interactions inherent to different solutions, may contribute to ensuring separation of the miscible liquids interacting within the liquid exchange unit.

Scanning electron microscopy (SEM) imaging revealed that the typical diameter of structures was between 20 and 50 µm. Interestingly, the diameter of a growth halted structure widens to around 100–150 µm toward a dome shaped apex (Fig. 2d). This microstructural evolution may result from the acceleration of calcium-rich fluid emitted from the tip into an environment of reducing density. As structural growth approaches the interface between phosphate solution and H_2_O, the flow of fluid emitted from the tips may be compressed due to the density of cationic fluid leaving the tip being relatively greater than that of H_2_O, leading to a downward swirling vortex akin to that seen in examples of Rayleigh-Taylor instability and Richtmyer-Meshkov instability^40,41^. Precipitation appears to capture the doming pattern of flow, resulting in the distinct tip morphology.

We next investigated whether the continued displacement of phosphate solution with H_2_O provides a means of purifying chemical garden structures in situ. Purification of products prepared by precipitation reactions is generally necessary in order to prevent the crystallisation of otherwise soluble phases on drying. X-ray diffraction (XRD) was used to examine the compositional purity of the calcium phosphate chemical gardens (Fig. 2f and see Supplementary information Figs. S1,  S2,  S3 and  S4). Following synthesis, tubular calcium phosphate structures that were harvested and then dried without purification were highly contaminated, with prominent diffraction peaks corresponding to contaminant salts of monoammonium phosphate (NH_4_H_2_PO_4_) and diammonium phosphate ((NH_4_)_2_HPO_4_) being apparent over those indicative of poorly crystalline calcium-deficient hydroxyapatite (CDHA) (Ca_9_HPO_4_(PO_4_)_5_OH). Purification of harvested structures with excess H_2_O and filtration apparatus eliminated these soluble contaminant phases, as shown by the appearance of peaks corresponding to CDHA only. Though effective for purification, we noted that it was very easy to damage chemical garden structures with this method. Alternatively, in situ purification was performed without compromising the structural integrity of calcium phosphate tubes and was in practice inherently more controllable than filtration. Chemical gardens were purified in situ with a volume displacement (VD) ratio of either 5:1 or 10:1 (H_2_O to phosphate solution) at a displacement rate of 10 mL/min. Contaminant phases were detectable when structures were purified with a VD of 5:1. Increasing the VD ratio to 10:1 resulted in structures that were comparably pure to those washed by filtration. Therefore, in situ purification employing a VD of 10:1 was effective at eliminating contaminant phases.

### Chemobrionic composite fabrication

We next explored the potential to utilise the liquid exchange unit for fabricating chemobrionic materials. It was envisioned that a chemobrionic composite incorporating highly aligned tubular channels could be engineered by displacing H_2_O following purification with either liquid polymers or hydrogels, which upon curing and/or cross-linking, respectively, would harden and encase the self-assembled structures within a surround matrix. In order to demonstrate the preparation of chemobrionic materials in principle, we prepared a chemobrionic composite using 2-hydroxyethyl methacrylate (HEMA), which forms a hydrogel when irradiated with UV light, as the matrix material.

**Fig. 3:**
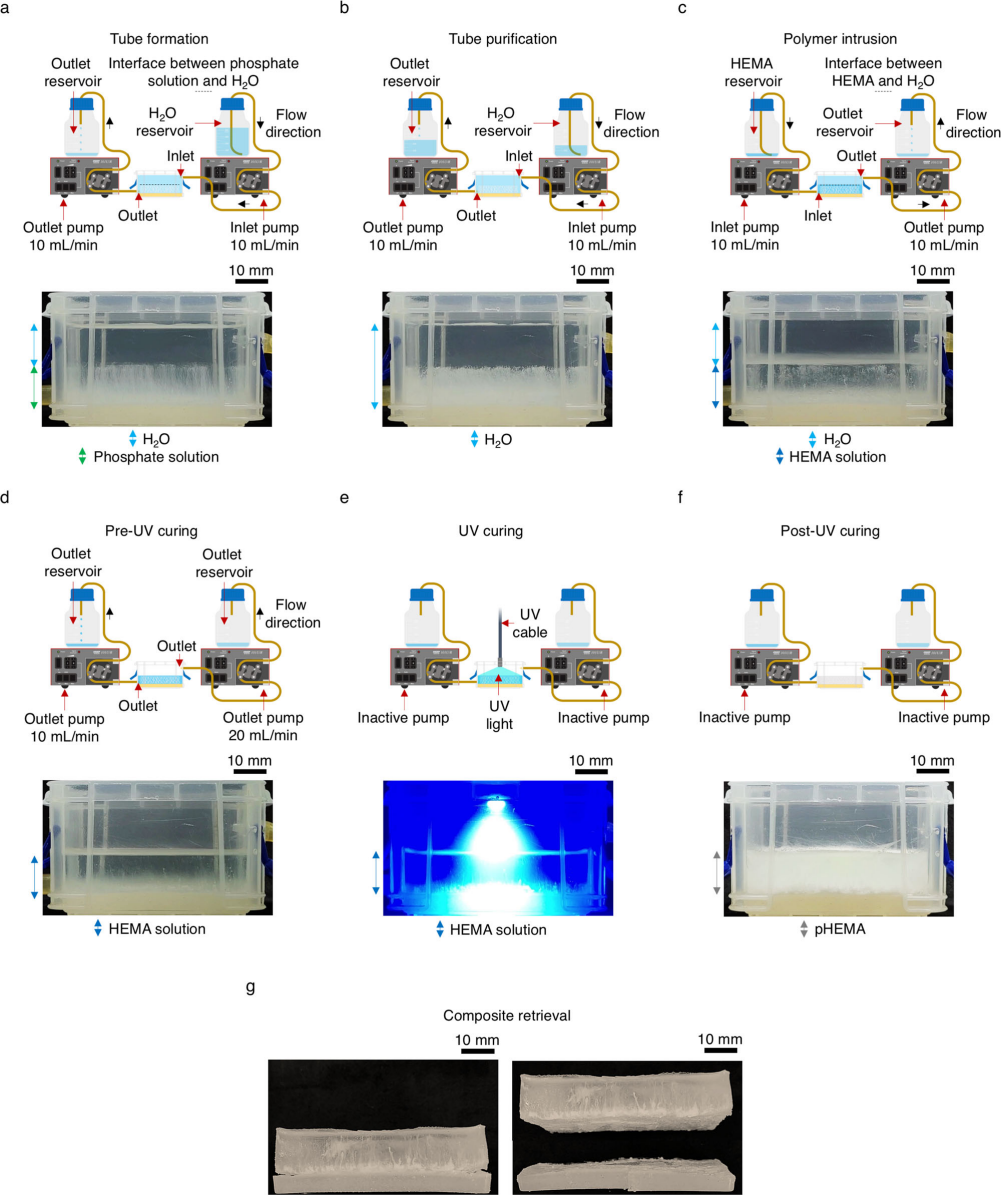
Fabrication of a chemobrionic composite incorporating self-assembled chemical garden structures. **a** Displacement of phosphate solution with H_2_O performed during the formation of calcium phosphate tubes. When the tips of the tubes met the established H_2_O layer, displacement was halted. **b** Structures purified in situ with a volume displacement (VD) ratio of 10:1 (H_2_O to phosphate solution). **c** Displacement of H_2_O with 2-hydroxyethyl methacrylate (HEMA) performed following purification. **d** Excess HEMA was drained until level with the tips of the growth halted calcium phosphate tubes. **e** UV irradiation of the liquid exchange unit and its contents. **f** UV irradiation encased the calcium phosphate tubes in a matrix of poly(2-hydroxyethyl methacrylate) (pHEMA). **g** Recovered contents of the liquid exchange unit. The composite was retrieved by peeling away the hydrogel layer.

Firstly, an array of calcium phosphate tubes was prepared (Fig. 3a). Displacement of phosphate solution with H_2_O was performed 10 s after the layering phosphate solution over the hydrogel at a rate of 10 mL/min. Displacement was halted when the tips of structures met the interface between phosphate solution and H_2_O. The calcium phosphate tubes were then purified in situ with a VD of 10:1 (H_2_O to phosphate solution) at a rate of 10 mL/min (Fig. 3b). Due to the density of HEMA (ρ = 1.06 ± 0.01 g/cm^3^ at 25 °C and 1 atm) being greater than that of H_2_O (ρ = 1.01 ± 0.01 g/cm^3^ at 25 °C and 1 atm), the conventional positions of the inlet and outlet channels were reversed prior to polymer infiltration. Displacement of H_2_O with HEMA was performed at a rate of 10 mL/min. Phase separation of the HEMA component was not observed during displacement, resulting in partitioning of the liquids, with H_2_O overlaying the incoming HEMA. Uncured HEMA was of a sufficiently low viscosity to permeate between individual tubes and completely surround the structures whilst maintaining alignment of the array (Fig. 3c). Following displacement of H_2_O with HEMA, excess HEMA was drained until level with the tips of the growth halted calcium phosphate tubes (Fig. 3d). To ensure complete removal of remaining H_2_O overlaying HEMA at this stage, displacement was performed at a rate of 10 mL/min at the lower outlet channel and at the 20 mL/min at the higher outlet channel. The contents of the liquid exchange unit were then irradiated with UV light for 10 min (Fig. 3e). Upon curing, the liquid HEMA cross-linked and strengthened, encasing the tubular structures in a pHEMA matrix (Fig. 3f). Following curing, the contents of the liquid exchange unit could be retrieved and composite material separated by peeling away the underlying hydrogel (Fig. 3g).

### Chemobrionic composite characterisation

**Fig. 4:**
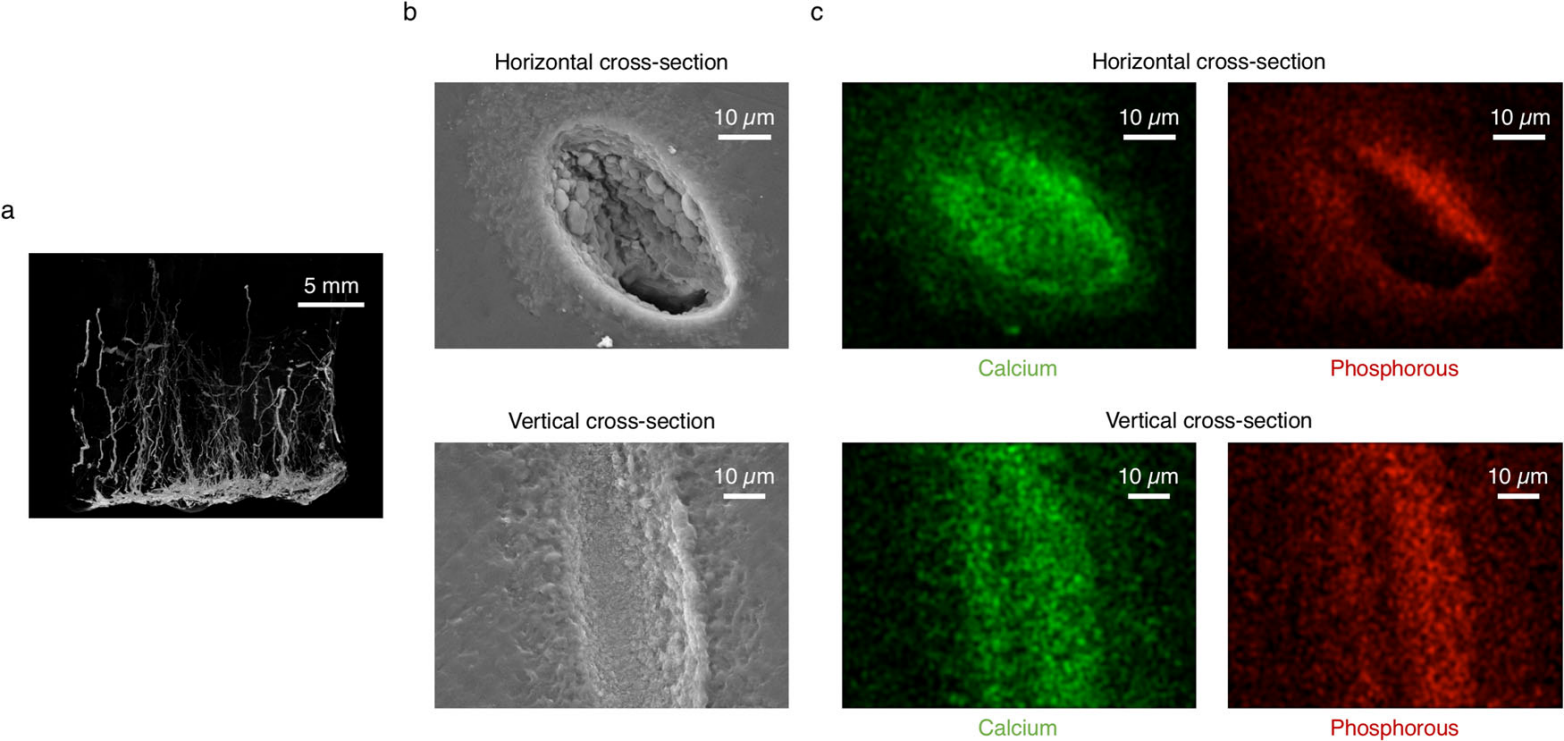
Microstructural characterisation of chemobrionic composite. **a** Micro-computed tomography (µ-CT) 3D reconstruction of a section of chemobrionic composite, which shows the alignment of calcium phosphate tubes encased within the poly(2-hydroxyethyl methacrylate) (pHEMA) matrix; scale bar = 5 mm. **b** Scanning electron microscopy (SEM) images of cross-sections of the chemobrionic composite along the horizontal and vertical planes, respectively, revealing intact tubular channels; scale bar = 10 µm. **c** Energy dispersive X-ray (EDX) spectroscopy elemental mapping, with elemental calcium and phosphorous coloured green and red, respectively, confirming the channels were composed of calcium phosphate; scale bar = 10 µm.

The retrieved composite material was sectioned prior to characterisation. Micro-computed tomography (µ-CT) 3D reconstructions revealed the relatively well-persevered vertical orientation of high-aspect-ratio channels throughout the bulk polymer (Fig. 4a). Sections of composite were also vacuum embedded in epoxy resin and abraded to attain cross-sections of the material across the horizontal and vertical planes. Subsequent SEM imaging of the respective cross-sections confirmed the presence of intact, hollow channels approximately 20–50 µm in diameter (Fig. 4b and see Supplementary information Figs. S5 and S6). Energy dispersive X-ray (EDX) spectroscopy elemental mapping further showed that the channels were composed predominantly of calcium and phosphorous species, indicative of the successful incorporation of biologically analogous chemical garden structures within the pHEMA matrix (Fig. 4c).

In relation to biomedical applications, pHEMA has previously been employed in the fabrication of bone repair composites in combination with calcium phosphate particles composed of hydroxyapatite (HA) and beta-tricalcium phosphate (ß-TCP) phases, which improve biocompatibility and integration with hard tissue^42,43^. The integration of structured calcium phosphate tubes provides seemingly perfusable channels in an otherwise dense matrix, not entirely dissimilar to Haversian canals enclosed in concentric layers of compact bone^44^. Tubular structures are inherent features of bone tissue that are vital for the supply of nutrients and oxygen, as well as waste removal^45^. Regenerative hard tissue scaffolds that possess tubular features that recapitulate microstructures found in bone are highly sought for improving cellular recruitment and tissue regeneration, as well as promoting implant vascularisation, beyond the integration of mineral particles and simple porosity^46,47^. In this respect, chemobrionic principles have the potential to significantly influence biomaterials design through the guided formation of heterogeneous systems and devices.

### Conclusion and outlook

In summary, the principles described herein demonstrate a significant leap toward controlling the formation chemical gardens that may further facilitate the fabrication of technologically relevant materials consisting entirely of, or incorporating, chemobrionic components. Whilst we have focused on controlling the formation of calcium phosphate chemical gardens, our approach could undoubtedly be adapted to influence the formation of structures grown from salt seeds or by injection methods, of which there are many compositionally diverse examples. Thus, as well as regenerative applications, there is also an opportunity to explore the generation of heterogeneous chemobrionic materials for a variety of uses, including catalysis and microfluidics. Future work will focus on refining the methods of composite fabrication, improving our understanding of the effects of  processing on the chemical garden structures and investigating the capacity of chemobrionic composites to direct biological healing processes.

## Methods

### Liquid exchange unit

The liquid exchange unit was engineered from a transparent 0.14 L “Really Useful Box” with external dimensions of 90 × 65 × 55 (length × width × height) and internal dimensions of 68 × 48 × 42 mm (length × width × height). Using a Dremel 3000 equipped with a 3.2 mm diameter high-speed cutter attachment (Dremel, USA), a total of two holes measuring 3.2 mm in diameter were introduced, each one centrally aligned on opposing sides of the box, positioned 25 mm and 5 mm from the base, respectively. Each hole was fitted with a small cone shaped piece of tubular plastic with an inward end diameter of 3.0 mm and an outward end diameter of 3.4 mm, which served as an adapter for the installation of flexible rubber tubing with an internal diameter of 3.4 mm and external diameter of 6.2 mm. Both pieces of tubing were fed through a set of Watson-Marlow101U/R peristaltic pumps (Watson-Marlow, UK) to enable the displacement of liquid phases from and to inlet and outlet reservoirs, respectively.

### Calcium phosphate chemical gardens

Calcium nitrate tetrahydrate (Ca(NO_3_)_2_.4H_2_O) (99%, ACS reagent), ammonium phosphate dibasic ((NH_4_)_2_HPO_4_) (≥98.0%, reagent grade) and agar (for microbiology) were acquired from Sigma-Aldrich (Sigma-Aldrich, UK). All mixtures and solutions were prepared with H_2_O with a recorded resistivity of 15–18 MΩ.cm (at 25 °C and 1 atm) from a Milli-Q^®^ Integral 3 water purification system equipped with Q-Gard^®^ and Quantum^®^ cartridges (Merck Millipore, USA). Calcium-loaded hydrogel was prepared by heating a mixture of Ca(NO_3_)_2_.4H_2_O ([Ca^2+^  = 1 M) and 5 w/v% agar to 80–90 °C whilst stirring at 250–500 rpm on a magnetic stirrer hot plate. Using a syringe, 15 mL of warm hydrogel mixture was cast evenly into the chemical garden liquid exchange unit by hand. The liquid exchange unit and its contents were then stored at 4 °C overnight to complete gelation before returning to room temperature. Self-assembly of tubular calcium phosphate frameworks was initiated by the addition of 85 mL phosphate solution prepared with (NH_4_)_2_HPO_4_ ([HPO_4_^2−^] = 1 M).

### Controlled formation and purification of chemical gardens

Displacement of phosphate solution with H_2_O was controlled by the operation of the peristaltic pumps. To visually demonstrate the interaction between phosphate solution and H_2_O in the absence of calcium-loaded hydrogel, the reactant reservoir was labelled with 2 µM fluorescein prepared from sodium fluorescein salt (Sigma-Aldrich, UK) prior to displacement. Complete displacement of reactant with excess H_2_O was performed as a means of purifying growth halted chemical garden structures. Reactant VD ratios of 5:1 or 10:1 (H_2_O to reactant) were employed to purify chemical garden structures (450 and 850 mL total volume displaced, respectively). In order to comparatively assess purification efficacy of this method against conventional approaches, structures were also prepared without washing or washing with excess H_2_O (500 mL) by filtration using Buchner funnel apparatus. Structures were then transferred to an oven and dried in air at 60 °C for 12 h.

### Chemobrionic composite fabrication

HEMA (97%, contains ≤250 ppm monomethyl ether hydroquinone as inhibitor), ethylene glycol dimethacrylate (EGDMA) (98%, contains 90–110 ppm monomethyl ether hydroquinone as inhibitor) and inhibitor removal beads were acquired from Sigma-Aldrich (Sigma-Aldrich, UK). Omnirad 4265 (mixture of Omnirad-TPO and Omnirad-73) was acquired from IGM Resins (IGM Resins, The Netherlands). To 60 mL of HEMA, 1 mL of EGDMA was added. To this mixture, 1 g of inhibitor removal beads was added whilst stirring at 250–500 rpm magnetic stirrer hot plate. After several minutes the beads were removed by filtration and 36 mL H_2_O was added, following which 1 mL of UV initiator (Omnirad 4265) was added whilst stirring at 250–500 rpm. The mixture was then deoxygenated by bubbling through nitrogen gas for 10 min. Following the growth and purification of calcium phosphate tubes, displacement of HEMA solution with H_2_O was performed and the excess HEMA drained. The liquid exchange unit and its contents were then irradiated at 20% irradiance for 10 min using an S2000 Spot UV Curing System (Excelitas Technologies, Canada). The contents of the liquid exchange unit were then removed and the composite separated from the underlying hydrogel. A scalpel was then used to section the chemobrionic composite prior to characterisation.

### Density measurements

The densities of phosphate solution, H_2_O and HEMA solution were determined by measuring the mass of 1 mL of liquid extruded from a calibrated Sartorius 100–1000 µL pipette (Sartorius, Germany) using a Quintix125D-1S microbalance (Sartorius, Germany). Measurements were performed in triplicate.

### X-ray diffraction (XRD)

Dried structures were powdered using a pestle and mortar before being transferred to an appropriate powder specimen holder. XRD was performed using a Diffractometer D8 autosampler instrument equipped with Cu Kα radiation (Bruker, USA). Diffraction patterns were acquired between 2θ = 5° and 60° with a step size of 2θ = 0.02° and step time of 0.5 s/°. The acquired patterns were then matched to patterns for known crystalline phases within the International Centre for Diffraction Data (ICDD) database using DIFFRAC.SUITE software (Bruker, USA).

### Scanning electron microscopy (SEM) and energy dispersion X-ray spectroscopy (EDX)

Growth halted structures were collected on a glass slide, gently washed with H_2_O and excess liquid removed using a pipette before drying in air at 60 °C for 12 h. Sections of the chemobrionic composite were embedded in EpoFix cold-setting embedding resin (Struers, UK) using a CitoVac vacuum embedding instrument (Struers, UK). To obtain horizontal and vertical cross-sections of the chemobrionic composite, embedded sections were abraded along respective planes, with progressively finer grit discs from FEPA P grade 120 to 4000 using a TechPrep grinder/polisher (Allied High Tech Products, USA). Prior to imaging, specimens were mounted onto aluminium stubs using double-sided carbon discs before being gold coated in argon atmosphere with the sputter current at 20 mA and coating time of 20 s using a K550X sputter coater (Quorum Technologies, UK). Embedded samples required the use of copper tape to ensure conductivity between the sample surface and the aluminium stub. SEM images and EDX elemental maps were acquired using a table-top TM 3030 Plus electron microscope coupled with an EDX detector operating with an electron acceleration voltage of 10–15 kV (Hitachi, Japan).

### Micro-computed tomography (µ-CT)

µ-CT was performed using a Skyscan1172 µ‐CT instrument (Bruker, USA). A section of chemobrionic composite was scanned with operating parameters of 80 kV source voltage, 100 µA current, 600 ms exposure time, 4 µm pixel size, rotation step of 0.1° and frame averaging of 20. The acquired slice data was reconstructed using NRecon software (Bruker, USA) and visualised using CTVox software (Bruker, USA).

## Supplementary information

Supplementary information.

## Data Availability

Datasets supporting the findings of the study can be requested from the corresponding author on reasonable request.
