# Supplementary material for: Controlled self-assembly of chemical gardens enables fabrication of heterogeneous chemobrionic materials
Source: Commun Chem. 2021 Oct 14;4:145. doi: 10.1038/s42004-021-00579-y (PMC9814108; doi:10.1038/s42004-021-00579-y)
Supplement: Supplementary file 1 — Supplementary information. [file 42004_2021_579_MOESM1_ESM.pdf]

Supplementary information

Controlled self-assembly of chemical gardens enables fabrication of heterogenous chemobrionic materials

[1,2]Erik A B Hughes*, [1]Thomas E Robinson, [1]Richard J A Moakes, [1]Miruna Chipara and [1]Liam M Grover

*Corresponding author: e.a.b.hughes@bham.ac.uk

[1]School of Chemical Engineering, University of Birmingham, B15 2TT, UK

[2]NIHR Surgical Reconstruction and Microbiology Research Centre, Queen Elizabeth Hospital, Birmingham, UK

Contents:

Fig. S1 Individual X-ray diffraction pattern for structures treated without purification with matched patterns from the International Centre for Diffraction Database (ICDD) displayed.

Fig. S2 Individual X-ray diffraction pattern for structures purified by filtration with excess H_2O with matched patterns from the International Centre for Diffraction Database (ICDD) displayed.

Fig. S3 Individual X-ray diffraction (XRD) pattern for structures purified *in situ* with a volume displacement (VD) ratio of 5:1 (H_2O to phosphate solution) with matched patterns from the International Centre for Diffraction Database (ICDD) displayed.

Fig. S4 Individual X-ray diffraction (XRD) pattern for structures purified *in situ* with a volume displacement (VD) ratio of 10:1 (H_2O to phosphate solution) with matched patterns from the International Centre for Diffraction Database (ICDD) displayed.

Fig. S5 Additional scanning electron microscopy (SEM) image of a cross-section of the chemobrionic composite along the horizontal plane, revealing intact tubular channels; scale bar = 30 μm.

Fig. S6 Additional scanning electron microscopy (SEM) image of a cross-section of the chemobrionic composite along the vertical plane, revealing intact tubular channels; scale bar = 30 μm.

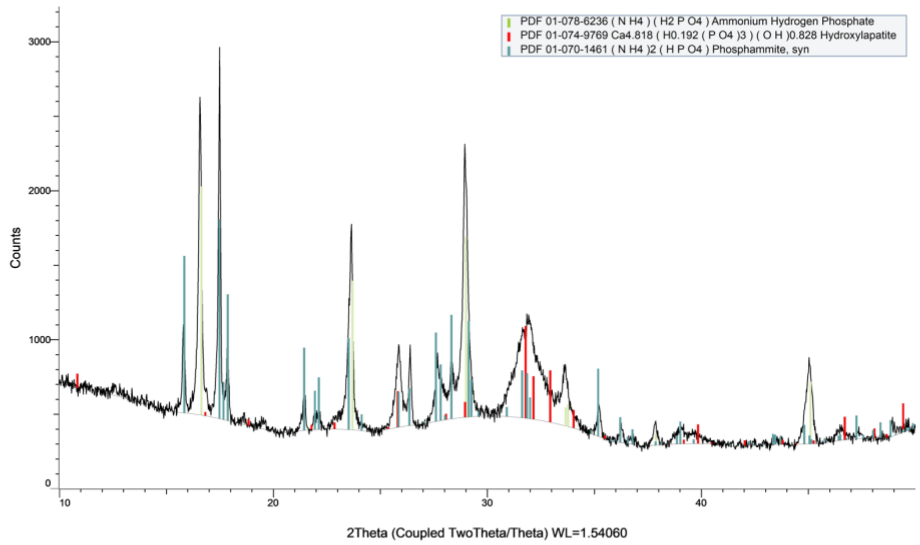

Fig. S1 Individual X-ray diffraction pattern for structures treated without purification with matched patterns from the International Centre for Diffraction Database (ICDD) displayed.

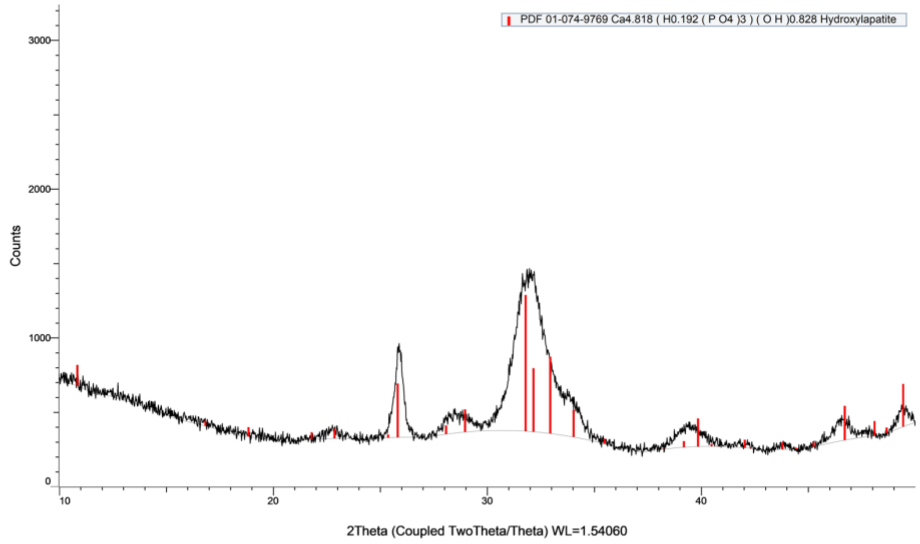

Fig. S2 Individual X-ray diffraction pattern for structures purified by filtration with excess H_2O with matched patterns from the International Centre for Diffraction Database (ICDD) displayed.

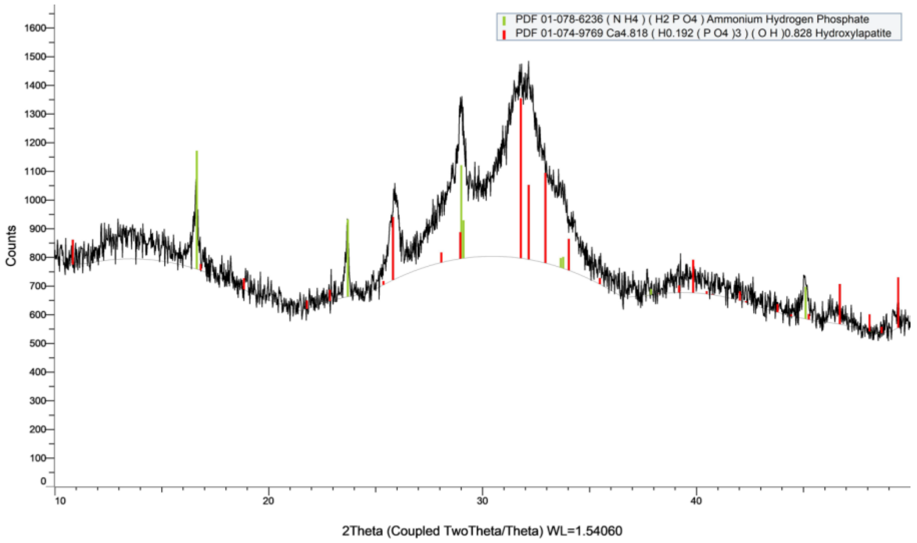

Fig. S3 Individual X-ray diffraction (XRD) pattern for structures purified *in situ* with a volume displacement (VD) ratio of 5:1 (H_2O to phosphate solution) with matched patterns from the International Centre for Diffraction Database (ICDD) displayed.

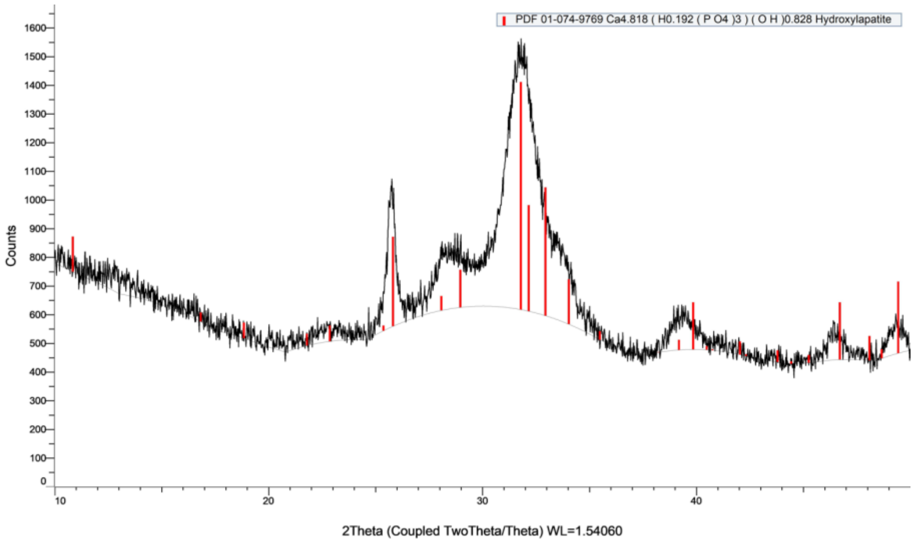

Fig. S4 Individual X-ray diffraction (XRD) pattern for structures purified *in situ* with a volume displacement (VD) ratio of 10:1 (H_2O to phosphate solution) with matched patterns from the International Centre for Diffraction Database (ICDD) displayed.

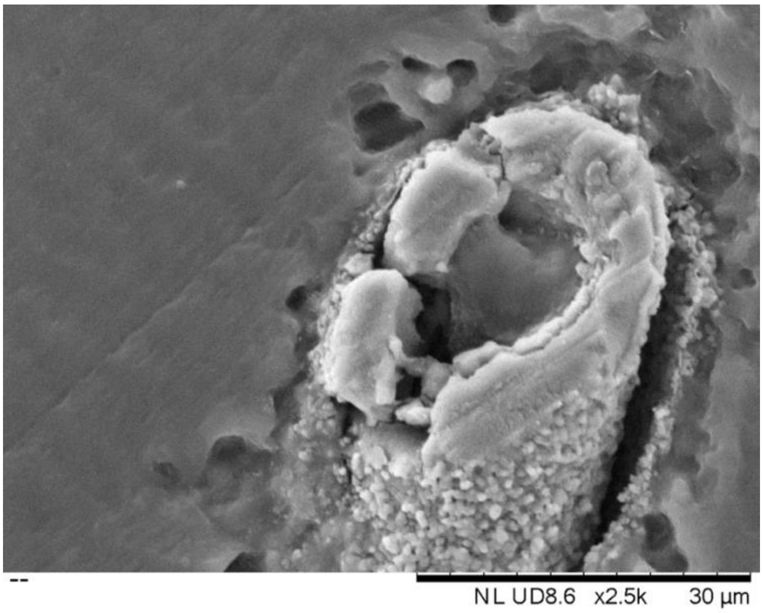

Fig. S5. Additional scanning electron microscopy (SEM) image of a cross-section of the chemobrionic composite along the horizontal plane, revealing intact tubular channels; scale bar = 30 μm.

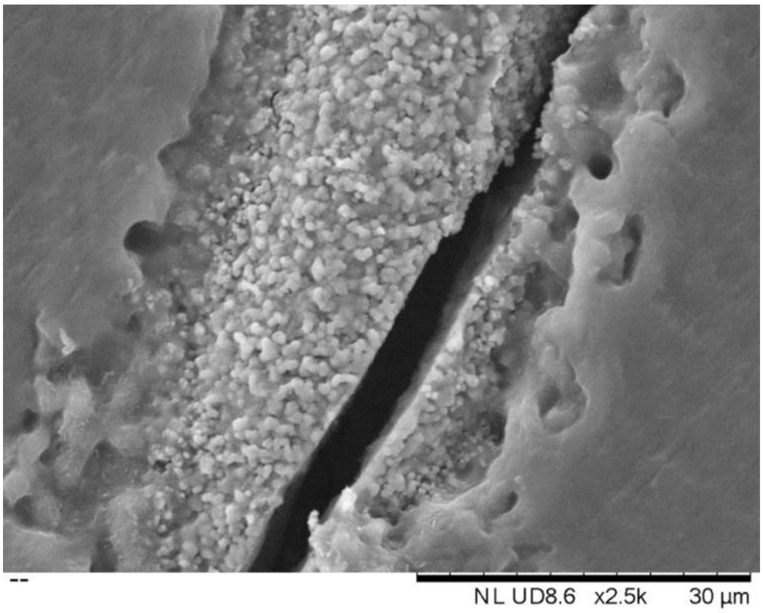

Fig. S6. Additional scanning electron microscopy (SEM) image of a cross-section of the chemobrionic composite along the vertical plane, revealing intact tubular channels; scale bar = 30 μm.
